# 'Linkage' pharmaceutical evergreening in Canada and Australia

**DOI:** 10.1186/1743-8462-4-8

**Published:** 2007-06-01

**Authors:** Thomas A Faunce, Joel Lexchin

**Affiliations:** 1 College of Law and Medical School, Australian National University, Canberra, Australia; 2 School of Health Policy and Management, York University, Toronto, Canada

## Abstract

'Evergreening' is not a formal concept of patent law. It is best understood as a social idea used to refer to the myriad ways in which pharmaceutical patent owners utilise the law and related regulatory processes to extend their high rent-earning intellectual monopoly privileges, particularly over highly profitable (either in total sales volume or price per unit) 'blockbuster' drugs. Thus, while the courts are an instrument frequently used by pharmaceutical brand name manufacturers to prolong their patent royalties, 'evergreening' is rarely mentioned explicitly by judges in patent protection cases. The term usually refers to threats made to competitors about a brand-name manufacturer's tactical use of pharmaceutical patents (including over uses, delivery systems and even packaging), not to extension of any particular patent over an active product ingredient. This article focuses in particular on the 'evergreening' potential of so-called 'linkage' provisions, imposed on the regulatory (safety, quality and efficacy) approval systems for generic pharmaceuticals of Canada and Australia, by specific articles in trade agreements with the US. These 'linkage' provisions have also recently appeared in the Korea-US Free Trade Agreement (KORUSFTA). They require such drug regulators to facilitate notification of, or even prevent, any potential patent infringement by a generic pharmaceutical manufacturer. This article explores the regulatory lessons to be learnt from Canada's and Australia's shared experience in terms of minimizing potential adverse impacts of such 'linkage evergreening' provisions on drug costs and thereby potentially on citizen's access to affordable, essential medicines.

## 'Linkage' Evergreening – Evolution from Hatch-Waxman to NAFTA

The 'linkage' form of patent 'evergreening' is an important strategy that multinational pharmaceutical companies have been using in the United States since the passage of the Waxman-Hatch legislation in 1984, to prolong rent-profits over 'blockbuster' (high total revenue) drugs. This legislation was originally designed to facilitate easier market entry for generic pharmaceuticals, in return for extending the patent term of brand name competitors as a partial compensation for delayed regulatory (safety, efficacy and quality) approvals. Quicker generic availability was perceived as profit-threatening by the brand-name pharmaceutical industry, chiefly because low cost, quality generics rapidly captured the bulk of market share after blockbuster patent expiry [1]. The legislation's promised grand compromise between access and innovation, however, failed to materialize, as provisions favorable to brand name manufacturers were progressively emphasized [2].

In 2002, an extensive and lengthy inquiry by the US Federal Trade Commission (FTC), found that the Waxman-Hatch legislation had resulted in as many as 75% of new drug applications by generic drug manufacturers experiencing legal actions under patent laws by the original brand name patent owner. These were driving up US drug costs by keeping the cheaper generic versions off the market. The FTC recommended that only one 'evergreening' injunction against a potential generic market entrant be permitted per product [3]. This complex 'anti-evergreening' legislative change (along with a process for expedited challenge) was implemented December 2003 under the *Medicare Prescription Drug Improvement and Modernisation Act 2003 (US)*. That legislation also implemented, however (after considerable industry lobbying) in the so-called Medicare Part D benefit, a fiscally expensive (adding $14 billion to Medicare costs by 2013) drug benefit subsidy (with no readily apparent upper limit of total expenditure) for those aged 65 and older, whilst preventing the Federal US Medicare agency from negotiating drug prices with drug companies on behalf of the multiple private Medicare Advantage drug plans which operate under Medicare Part D [4].

In 1993, under the *North American Free Trade Agreement *(NAFTA)-induced Canadian *Notice Of Compliance *(NOC) 'linkage' regulations, drug safety, quality and efficacy regulators at Health Canada were prevented from issuing an authorization for market entry, until the all of the relevant patents on a brand name product had been proven to have expired. As a result, when a Canadian generic company (such as Apotex) submits its application to get a product approved, it also sends a Notice of Allegation (NOA) to the patent holder claiming that no patents are being infringed. The patent holder then has 45 days in which to initiate an application in the Federal Court of Canada, seeking an order to prohibit the relevant Minister from issuing a Notice of Compliance to the generic manufacturer for a period of 24 months, or upon resolution of the court application, whichever is sooner.

The NOC 'linkage' regulations, were formulated by Industry Canada and are administered by the Office of Patented Medicines and Liaison, located in the Therapeutic Products Directorate, Health Products and Foods Branch, Health Canada. They require the Minister of Health to maintain a Patent Register [5]. This consists of patent lists submitted in respect of eligible NOC-issued drugs. The Minister responsible for Health Canada may refuse to add, or may delete, information from this Patent Register. Each patent list is audited (for example as to whether potential inclusions are mere 'evergreening' attempts) by the Office of Patented Medicines and Liaison. Reports produced by that body outline statistics relating to the maintenance of the Patent Register, including the number of patents filed, the number of patents accepted and rejected, and litigation resulting from the acceptance or rejection of patents for listing on the Patent Register. They also include statistics on the outcomes of generic drug manufacturers filing a Form V Patent Declaration with the Minister, and serving an NOA on the relevant patented drug manufacturer [6].

The effect of these 'linkage' regulations has been a subject of intense disagreement between the Canadian generic and brand name companies. The Canadian Generic Pharmaceutical Association (CGPA) claims that "not only is this abuse of Canada's patent regime extremely harmful to Canada's generic pharmaceutical industry, the Canadian public loses out on millions of dollars in savings by having to pay for the higher-priced brand-name version for an extended period of time. The delays caused by these needless court battles have cost Canadians, their governments and private insurers hundreds of millions of dollars." [7]. The increase in drug spending attendant on the delay in the appearance of generic equivalents may partly explain why provincial governments have increased co-payments and deductibles for recipients of publicly funded drug plans.

Canada's Research-Based Pharmaceutical Companies (Rx&D), the peak brand name industry association, counters that these regulations are necessary because "generics do not have to concern themselves with a possible interlocutory injunction to prevent infringing sales once an infringing generic product is on the market. Statistics show that this remedy is available in pharmaceutical cases approximately half as often as in other industry patent cases. Indeed, as a result of the inability of pharmaceutical patentees to obtain interlocutory injunctions to prevent the complete destruction of their intellectual property rights and market share, the 'linkage' regulations are the only means for Canada to meet its international obligations to provide an effective enforcement mechanism for patents."[8]. In-other-words, Rx&D's claim is that the availability of injunctions against the marketing of a generic product is insufficient protection because these injunctions are frequently unavailable to the brand-name companies.

Rx&D also points out that the approximate 80% success rate for the generic companies, translates into 4 out of 5 'anti-evergreening' cases won and presents its own figures. The way that Rx&D calculates the judicial outcomes appears to show a roughly equal split in wins. However, an examination of this figure reveals that the brand name companies are also not above playing around with numbers. There are 125 cases where there was no hearing, in 20 cases where the NOA was withdrawn this is counted as a win for the patentee but the 100 cases where the innovator either accepted the NOA or the case was otherwise settled are not counted as wins for the generic [9].

A particularly contentious related tactic is the use of multiple patents to delay the appearance of a generic product. The CGPA maintains that the brand name companies continually list new patents on a product, each of which can trigger a new NOA and an additional stay on the appearance of a generic. In this way, competition is delayed [10]. The brand name companies dispute this interpretation. Their position is that there is always ongoing research into drugs and that it is natural that new patents will be filed, reflecting improvements such as moving from a three pill a day regimen to once a day dosing. Multiple patents on a single medicine are relatively common.

The Office of Patented Medicines and Liaison at the Therapeutic Products Directorate of Health Canada estimates that 44% of the 419 medicines on the Patent Register are covered by more than one patent [11]. The multinationals say that 95% of cases all subsequent patents will be issued within 10 years of the initial patent and therefore all of the patents may be addressed in the same linkage proceeding. But if the effective patent life is only 10 years, as Rx&D claims it is then new patents are being filed as old ones expire. Even if patents are a couple of years longer then there can still be overlapping 24 month stays depending on when the generic company files for a NOC.

Between 1998 and 2004, out of 138 cases that have gone to court 12% have taken more than 24 months to resolve [12]. According to Rx&D in that situation all the generic companies have to do is market the older version of the product on expiry of the original patent [13]. All of this is true but it ignores the fact that the main reason for launching a new formulation of a drug is to switch doctors to prescribing that version before a generic is available to undercut the market. This is a form of 'evergreening' that brand name companies spend millions of advertising dollars doing.

In October 2006, the Canadian federal government recognized that some brand-name companies had been abusing the NOC Regulations. It limited their use of 'evergreening' follow-on patents by promulgating regulations that prevented any new patents they filed after a generic company had submitted an application for approval of its product from being considered in the NOC Regulations process. Moreover the new regulations made it clear that patents covering areas without direct therapeutic application, such as processes or intermediates, could not be used to delay generic approval [14]. Less than two months later the Supreme Court of Canada also recognized that the brand-name companies had been abusing the NOC Regulations by adding irrelevant patents [15].

Canadian and US regulatory authorities are now being choked by an overgrowth of additional brand name medicine patent 'evergreening' tactics extending far beyond those implicit in these 'linkage' provisions. These include miniscule modifications in delivery system or formulation, combinations, licensing agreements ('authorised generics') and 'buy-outs' of generic competitors. The US Food and Drug Administration (FDA), for example, was recently asked to approve the marketing of Pfizer's new drug, called torcetrapib, which increases lipid-removing (or HDL) cholesterol, only in combination with Pfizer's high sales volume 'blockbuster' Lipitor (that lowers LDL cholesterol). Lipitor, which loses its original patent protection in 2010, is the world's top-selling medicine, with sales of almost $11 billion last year. If Pfizer's "evergreening" combination is backed by plausible research, the FDA may have to approve it, protecting Pfizer from actions under antitrust laws. Patients, however, who cannot tolerate (or afford) Lipitor, will be unable to obtain torcetrapib for use with another statin. Physicians wishing to raise a patient's level of HDL cholesterol – but who do not want to be forced to use Lipitor – will not have access to torcetrapib [16]. Pfizer, in fact, later announced it would sell torcetrapib on its own and in December 2006 halted development of torcetrapib entirely following results of a clinical trial showing that patients who took the drug had an increased risk of cardiovascular mortality [17].

In summary, although 'evergreening' encompasses a wide variety of tactics, the 'linkage' form this article concentrates on has evolved considerably from the Waxman-Hatch legislation and was inserted in NAFTA. In essence it requires that laws be enacted requiring generic manufacturers to notify brand name competitors of their intention to enter the market. Such laws also require government drug quality, safety and efficacy regulators link marketing approval for a generic medicine with the absence of patent claims. Having received notification of a possible market entry by a generic product, a threatened brand-name manufacturer then seeks to dissuade such competition, for example, by claiming what are sometimes large numbers of complex and often highly speculative patents covering the packaging or delivery system of the drug, instead of its active product ingredient [18]. Where a generic manufacturer has limited resources, such a threat of patent litigation is often enough to induce its managing directors to remove a drug from application. Even if the generic manufacturer is sufficiently funded and motivated (like Apotex in Canada) to call the litigation bluff, the brand name owner enjoys sustained sales from its blockbuster till all patent proceedings are completed.

## 'Linkage' Evergreening and the AUSFTA

Pharmaceutical 'evergreening' had received only brief public, judicial and regulatory attention in Australia prior to the AUSFTA. The 2002 High Court case of *Aktiebolaget Hassle v Alphapharm Pty Limited *[19] was one such example. It concerned a pharmaceutical patent owned by the Astra Group, over an oral pharmaceutical preparation in the form of a tablet, capsule or pellet containing omeprazole as the active ingredient. In 1998, as the term of the compound patent neared its end, Alphapharm, a generic manufacturer, commenced steps to import and sell in Australia a pharmaceutical preparation containing omeprazole for therapeutic use in the treatment of gastrointestinal diseases. It applied to the TGA to import and market in Australia such a pharmaceutical preparation. In August1998, Astra instituted a proceeding in the Federal Court to restrain apprehended infringement of each of the claims of the Patent and for other relief. Justice Kirby in his judgment stated:

The strategies that large pharmaceutical manufacturers have employed to avoid such generic competition, which include the use of intellectual property law, have been detailed elsewhere. They have attracted the attention and response of the Federal Trade Commission in the United States. Such battles have had their counterparts in many other countries. They present serious issues for the developing world...In its interpretation of the legislation, and in identifying the proper approach to the ultimately factual determination of obviousness called for by that statute, this Court should avoid creating fail-safe opportunities for unwarranted extensions of monopoly protection that are not clearly sustained by law [20].

In 2004 *Arrow Pharmaceuticals Limited v Merck & Co., Inc *[21] came before Gyles J in the Federal Court involving an application by Arrow for revocation of a number of claims of a patent granted to Merck. In the opening paragraph of his judgment, Giles J noted that ' [t]he case involves what would now colloquially be called an attempt to 'evergreen' a pharmaceutical patent'. Giles J based this interpretation on the substantial evidence before the court relating to the research and development process undertaken at Merck and the significant input of Merck's marketing department into decisions regarding research and development [22]. The decision was appealed and the appeal heard by the Full Court in May 2005. The majority said little about evergreening [23].

In 2004, however, in the late stages of public and legislative debate over the Australia-US Free Trade Agreement (AUSFTA) it became clear that one of its provisions, once incorporated into Australian law, might facilitate the process of pharmaceutical patent prolongation which has become known as 'linkage evergreening.' Article 17.10.4 of the AUSFTA required that Australia's Therapeutic Goods Administration (TGA) create a process where by a brand name manufacturer would be informed of an intended generic product and that marketing approval on safety and quality grounds be "prevented" wherever a competing product was "claimed."

Article 17.10.4 of the AUSFTA provided that:

Where a Party permits, as a condition of approving the marketing of a pharmaceutical product, persons, other than the person originally submitting the safety or efficacy information, to rely on evidence or information concerning the safety or efficacy of a product that was previously approved, such as evidence of prior marketing approval by the Party or in another territory:

(a) that Party shall provide measures in its marketing approval process to **prevent **those other persons from:

(i) marketing a product, where that product is **claimed **in a patent; or

(ii) marketing a product for an approved use, where that approved use is **claimed **in a patent,

during the term of that patent, unless by consent or acquiescence of the patent owner; and

(b) if the Party permits a third person to request marketing approval to enter the market with:

(i) a product during the term of a patent identified as claiming the product; or

(ii) a product for an approved use, during the term of a patent identified as claiming that approved use,

the Party shall provide for the patent owner to be **notified **of such request and the identity of any such other person. [emphasis added]

This provision linked, for the first time in Australia, the marketing approval regulatory process for generic pharmaceuticals (on clinical quality, safety and efficacy grounds), with their patent infringement status. Its formulation was clearly derived from the previously discussed Waxman-Hatch legislation introduced in the US in 1984 and the Canadian NOC Regulations implemented after NAFTA in 1993 [24]. Article 17.10.4 (a) did not define either "prevent" or "claimed," though an interpretation of these terms may be gained from implementing legislation passed by the Australian parliament as a condition of entry into force of the AUSFTA. In addition, AUSFTA 17.10.4 (b) required that the patent holder be notified of any generic marketing approval application.

Australian negotiators had stated to a federal Senate Inquiry into the impact of the AUSFTA that their legitimate expectation was that article 17.10.4 would not lead to 'evergreening' [25]. Other commentators disagreed [26]. They considered that the clear aim was to provide multinational (US) pharmaceutical patent holders with a mechanism for using the Australian judicial system to protect the life-span of their intellectual property rent from generic competition [27].

The Commonwealth of Australia Senate Select Committee did find that the AUSFTA final text may not have gone as far down the pro-pharmaceutical industry road as US negotiators were originally pushing for. Its final report also warmly endorsed the Australian legitimate 'anti-evergreening' expectations behind the TGA implementing legislation and supported legislative penalties for 'evergreening' and capacity of the Commonwealth Attorney General to join any action and reclaim taxpayer monies lost to 'evergreening'. The Committee's conclusion was that ' [a]ny delay to the marketing of generic drugs as a consequence of these changes, however slight, will have a cost to the PBS, state governments and consumers'. It also encouraged research to actively monitor the impact of these changes [28].

Legislatively fulfilling the article 17.10.4 obligation, as mentioned, was a requirement of entry into force of the AUSFTA, and Australia had to enact consequent amendments to the *Therapeutic Goods Act 1989 *(Cth). The amendments inserted a new section 26B which required applicants for marketing approval to certify that their product would not infringe a valid patent claim, or that the patent holder has been notified of the application.

However, after vigorous public debate, the implementing legislation went further and also introduced a new section 26C. This provides that where a certificate has been given under s26B by a generic manufacturer and the patent holder wishes to claim a patent and institute infringement proceedings, he or she must first certify that the proceedings are being commenced in good faith, have reasonable prospects of success (as defined in s26C(4)) and will be conducted without unreasonable delay. If the certificate is found to be false or midleading, fines of up to $10 million apply and the Cth Attorney General is permitted to join the action to recoup losses to the PBS. Section 26D provides that a patent holder who seek an interlocutory injunction to prevent the marketing of the generic pharmaceutical must obtain leave from the government to do.

Sections 26C and 26D are the so-called 'anti-evergreening' provisions, designed to prevent patent holders from manipulating the court system to lengthen the term of the patent and delay the entry of generic pharmaceuticals into the market. They are a strong statement of Australia's 'legitimate expectations' of benefit (freedom from pharmaceutical price rises due to 'evergreening') in this area. They will be particularly important in countering claims (based on Annex 2C.1 of the AUSFTA) that in implementing this pharmaceutical policy Australia has not been adequately 'valuing innovation.' This provision is a 'constructive ambiguity' as the Parties failed to conclusively define the term 'innovation', or to agree on a conclusive mechanism for its determination. The Australian negotiators favoured valuing pharmaceutical innvovation through 'objectively demonstrated therapeutic significance' under mechanisms such as PBS cost-effectivness analysis and reference pricing once that has lead to a cost-minimisation conclusion. Their US counterparts, on the other hand, supported the valuing of innovation through the operation of markets that are at least nominally 'competitive'.

Ensuring that 'evergreening' does not impede rapid market entry for generic medicines on brand name patent expiry, represents a crucial underlying precondition to the continuance of low prices in the PBS medicines cost-effectiveness pricing system. Australian negotiators made clear that existing mechanisms of evidence-based pharmacoeconomic analysis and reference pricing in a unitary formulary were to be retained after the AUSFTA [29]. As Australia's chief AUSFTA negotiator stated before the special Senate roundtable on the PBS and the AUSFTA:

We are not importing the Hatch-Waxman legislation into Australian law as a result of the free trade agreement... [Article 17.10.4] will not extend the time of the marketing approval process, and it does not add or provide any additional rights to the patent holders in that process...there is no injunction that can be applied under this article...it will be clear in the legislation tomorrow....we are establishing a measure in the marketing approval process that will fully meet the commitments under this article [30].

The US, nevertheless, has expressly signalled their disapproval of Australia's implementation of article 17.10.4 in an exchange of letters between the Australian Minister for Trade and the US Trade Representative on the implementation of the AUSFTA, in which the USTR stated:

If Australia's law is not sufficient to prevent the marketing of a product, or a product for an approved use, where the produce or use is covered by a patent, Australia will have acted inconsistently with the Agreement. We will be monitoring the matter closely, and reserve all rights and remedies as discussed below.

We also remain concerned about recent amendments to sections 26B(1)(a), 26C and 26D of the Therapeutic Goods Act of 1989. Under these amendments, pharmaceutical patents owners risk incurring significant penalties when they seek to enforce their patent rights. These provisions impose a potentially significant, unjustifiable, and discrimintory burden on the enjoyment of patent rights, specifically on owners of pharmaceutical patents. I urge the Australian Government to review this matter, particularly in light of Australia's international legal obligations. The United States reserves its rights to challenge the consistency of these amendments with such obligations [31].

The US has achieved a similar provision to article 17.10.4 of the AUSFTA in article 18.9.4 of the Korean-United States Free Trade Agreement (KORUSFTA). Article 18.9.4 of the KORUSFTA provides:

Where a Party permits, as a condition of approving the marketing of a pharmaceutical product, persons, other than the person originally submitting safety or efficacy information, to rely on that information or on evidence of safety or efficacy information of a product that was previously approved, such as evidence of prior marketing approval in the territory of the Party or in another territory, that Party shall:

(a) provide that the patent owner shall be notified of the identity of any such other person that requests marketing approval to enter the market during the term of a patent **notified to the approving authority **as covering that product or its approved method of use; and

(b) implement measures in its marketing approval process to **prevent **such other persons from marketing a product without the consent or acquiescence of the patent owner during the term of a patent notified to the approving authority as covering that product or its approved method of use [32].

The significant advance (in terms of facilitating rapid and plentiful generic market entry) of article 18.9.4 of the KORUSFTA over article 17.10.4 of the AUSFTA, is that for the notification process to commence the patent holder must first have notified the safety and efficacy regulator. This facilitates the creation of a specific list of approved pharmaceutical patents, notification not being required to the owner of nay patents on the list. It also encourages creation of a regulatory oversight agency capable of winnowing out mere 'me-too' pseudo or 'evergreening'-patents.

To Canadians observing these developments there must have been a significant element of déjà vu. In 1993, as mentioned, NAFTA had required the Canadians to implement a similar process.

## Lessons from Canada's and Australia's Response to 'Linkage' Evergreening

Canada appears to have benefited from directly confronting the threat of 'linkage evergreening' and implementing regulatory processes designed to minimize it. Australian authorities, on the other hand, are reluctant to admit that pharmaceutical 'linkage evergreening' exists, possibly because of the embarrassment such recognition might cause in relation to the way they may have conducted the AUSFTA negotiating process on such issues. Medicines Australia, the Australian brand name pharmaceutical industry lobbying body, has denied that 'evergreening' occurs in Australia.

Evergreening is a foreign practice with no relevance in Australia. It will not be allowed to occur in Australia due to our intellectual property laws and because frivolous patents fail by definition [33].

Such ebullient statements are characteristic of the 'spin' issuing from this patent pharmaceutical industry lobby group. They studiously neglect to discuss any similarities between the terms and purposes of article 17.10.4 of the AUSFTA, and the US and Canadian 'linkage' evergreening' provisions, as well as the regulatory and public health problems which result.

In Canada, the Office of Patented Medicines and Liaison under Health Canada has become an important regulatory mechanism for policing 'linkage' evergreening. No attempt has been made to create a similar multidisciplinary regulatory agency in Australia. Yet, it appears that article 18.9.4 of the KORUSFTA has been specifically drafted to permit the establishment of such a pharmaceutical patent 'anti-evergreening' oversight agency.

In Canada, brand name pharmaceutical manufacturers have become increasingly concerned that while the NOC regulations could provide the "basis for effective protection of pharmaceutical patent owners' rights as required under TRIPS and NAFTA. . . experience suggests that Health Canada is taking steps to avoid the necessary application of the regulations." [34]. Among other things, the US Pharmaceutical Research and Development Association (PhRMA) claims that Health Canada has been inconsistent in its policies and practices relating to the listing and delisting of brand name companies' patents and in requiring generic companies to send a NOA. They claim that Health Canada is continually and systematically limiting further the types of patents that can be listed on the Patent Register, that Canadian courts fail to provide effective recourse in cases where an NOC is issued for an infringing generic medicine and that, ultimately, Canadian courts are not applying standards required of them under NAFTA and TRIPS. PhRMA's ultimate conclusion is that the "USTR should attach high priority to remedying this situation."

Canada's compliance with its related TRIPS and NAFTA obligations remains a problem for PhRMA. Although Canada has instituted statutory data protection, several judicial rulings have cast doubt on enforcement, as required by TRIPS Article 39.3 and NAFTA Article 1711 [35]. The Canadian government appears to have buckled industry criticism of its inadequate so-called 'TRIPs-Plus' ['TRIPS-minus' from a public health point of view] protection of data exclusivity. This fails to understand that patents are really an intellectual monopoly privilege granted in return for community access to data. Data exclusivity prevents data submitted as part of the drug regulatory approval process from being made available to generic competitors to facilitate 'springboarding' or early market entry on patent expiry. At the same time that the Canadian government moved to limit the use of follow-on patents by the brand-name patented pharmaceutical companies, it also granted them an 8 year period of data exclusivity [36].

In fact, data exclusivity provisions, permitting periods of protection potentially cumulating for multiple uses and potentially blocking even compulsory licenses, appears to have dangerous (in terms of access to essential medicines) synergies with 'linkage evergreening' structures. One lesson here may be that regulatory data exclusivity be limited to pharmaceuticals that have been assessed by experts to be truly innovative, be restricted from cumulative applications and be prohibited from 'evergreening' an otherwise expired patent term.

Canada's experience is another piece of evidence that a trade dispute with the US over 'linkage evergreening' requirements remains a strong possibility in Australia. U.S. Trade Representative Robert Zoellick, as earlier mentioned, in his exchange of letters with Trade Minister Vaile expressly reserved the right of the U.S. to call into question Australia's linkage and anti- evergreening amendments to the TGA Act [37].

In the concluding paragraphs of this letter the U.S. Trade Representative stated:

bringing the Agreement into effect is without prejudice to any future action the US Government may take regarding compliance of Australia's laws and other measures with the Agreement ... [i]f subsequent practice reveals problems with the full exercise of US rights I have discussed above, Australia should expect that we will take appropriate remedial action.

Indeed late in 2005 the US Ambassador flagged that if a practical problem did emerge in the operation of these anti-evergreening provisions, which the countries had temporarily 'agreed to disagree' on, then the U.S. would first approach Australia for a bilateral resolution, but failing that would litigate the matter before the WTO [38]. It is unlikely because of the entrenched industry interests involved, that such a dispute will be resolved at the consultation stage. It is also unlikely that the US would use the AUSFTA choice of forum provision (article 21.4) to use a WTO panel before which its chances would be less strong. The issue could become critical for Australia if it passes legislation (*National Health Amendment (Pharmaceutical Benefits Scheme) Bill 2007 (Cth)) *fracturing its unitary PBS formulary into an F1 category (for patented medicines) and an F2 class (for generic medicines subject to mandatory price reductions). With reduced profit margins in a low volume market, generic companies in Australia (likely to be mostly subsidiaries of foreign firms-witness the recent takeover of Alphapharm by US Mylan Laboratories) will be in reduced position to challenge evergreening claims, contribute to significantly reduced public expenditure on medicines, or to take up the national interest challenge of compulsory licensing in public health emergencies.

A brief examination of the dispute resolution experience under NAFTA suggests that any process of appointing AUSFTA panel members can be protracted and susceptible to diplomatic pressure [39]. The AUSFTA provisions regarding the appointment of panel members resemble but are not identical to those found in the NAFTA and the Dispute Settlement Understanding (DSU) of the WTO [40]. Under the WTO DSU panelists are usually non-Party members of WTO delegations or academics and are also not nationals of the Parties unless by specific agreement [41]. They are generally expected to have expertise relevant to to the dispute [42]. In the ten years between the ratification of NAFTA in 1993 and 2003, the NAFTA countries were unable to fully agree on the roster of pre-approved panellists [43]. In the first two disputes to require the establishment of a panel under the NAFTA, the process of selecting a panel chair took six months [44]. Law professors seem to dominate amongst persons chosen to be NAFTA panelists [45].

Some might argue that Australia may have prepared itself slightly better than Canada for a potential trade dispute over the 'linkage evergreening' provisions. Amongst the AUSFTA-provoked amendments to the Australian *Therapeutic Goods Act 1989 *were provisions (the new ss26C and 26D) allowing, amongst other things, the Australian Federal Attorney-General to join an application for an injunction by a brand name patent holder against a generic medicines manufacturer and to claim damages where the injunction has caused a price rise under the Pharmaceutical Benefits Scheme (PBS). They may result in more scrutiny being applied by a patentee to the merits of its case, in particular in relation to which of its claims it will seek remedies for.

The Australian 'anti-evergreening' legislation may best be viewed as analogous to a unilateral interpretive declaration (something reasonably common in US bilateral treaties as a result of US Senate oversight). It sets out Australia's legitimate expectations that the benefit of US acceptance that the fundamental architecture of the PBS cost-effectiveness system (scientific evaluation of pharmaceutical innovation in terms of community benefit) would not be affected and that no 'linkage evergreening' system would be imported into Australia (to raise medicines patent rents or prices) via article 17.10.4 of the AUSFTA.

The [Australian] Government's agreement to increased transparency and a review process is consistent with current PBS legislation. No change is required to that legislation to effect our commitment to the US under this agreement [46].

The Government of Australia retains the right and authority to set the prices of medicine under the PBS. The provisions of the pharmaceutical annex to the Agreement will help improve market access for pharmaceuticals in Australia by improving the transparency and accountability of Australia's PBS system [47].

We have negotiated an outcome in 17.10.4 and in other areas of the agreement that will not lead to the delay of generic medicines into this country...We are not importing the Hatch-Waxman legislation into Australian law as a result of the free trade agreement [48].

Both the International Federation of Pharmaceutical Manufacturing Associations and the US PhRMA have reportedly commented that their view is that these provisions are inconsistent with obligations under TRIPS article 27 prohibiting discrimination in an area of technology (pharmaceuticals) [49]. The US is likely to argue, in this context, that the Australian anti-'linkage evergreening' legislation affects only pharmaceutical patents and is therefore discriminatory. Australia, on the other hand, could argue that where a unique problem arises specifically referable only to a particular field of technology, a solution applying *sui generis *only to that field of technology cannot be said to be discriminatory. This definition of discrimination is in accordance with the ordinary meaning and purpose of the TRIPs agreement.

In the *Canada – Patent Protection of Pharmaceutical Products *case, for example, Australia argued that discrimination in relation to enjoyment of patent rights should be distinguished from the application of uniform rules in all areas of technology. Further, Australia argued that it was not inconsistent with TRIPS to provide for distinct patent rules that responded to practical consequences of differences between fields of technology [50]. An illustration of this point is the obligations of disclosure for inventions which are micro-organisms, in which inventors must deposit a sample of the micro-organisms in accordance with the Budapest Convention. This method of disclosure is unique to inventors of micro-organisms, but it is not discriminatory because the issue only arises with respect to micro-organisms.

In fact, there are a number of obligations imposed by the AUSFTA that relate to the enjoyment of patent rights for pharmaceuticals alone, including extension of the terms of a pharmaceutical patent to compensate the patent owner for unreasonable curtailment of the effective patent term as a result of the marketing approval process (17.9.8(b)). This is clearly not discriminatory because the issue of delays in enjoyment of patent rights due to the marketing approval process arises only in the context of pharmaceutical patents.

To hold that sections 26C and 26D are discriminatory as to the field of technology would necessitate finding that the whole of Chapter 6, Part 3 of the *Patents Act 1990 *(Cth) dealing with extensions of patent terms for pharmaceutical patents is also discriminatory and in breach of Australia's TRIPs obligations. The simplistic definition of discrimination that gives rise to such a result cannot be said to in accordance with the ordinary meaning of the term, in light of the object and purpose of the TRIPs agreement, or the AUSFTA under article 21.9.2 (incorporating articles 31 and 32 of the VCLT). There could be many important lessons here for other nations dealing with 'linkage' 'evergreening' imposed through trade agreements

One particular area of interest in any such 'linkage evergreening' trade dispute would be the role of non violation nullification of benefits (NVNB) remedies. Article 21.2(c) of the AUSFTA, for example, provides that the dispute settlement provisions of the agreement may be initiated where a Party considers that:

[A] benefit the Party could reasonably have expected to accrue to it under Chapters Two (National Treatment and Market Access for Goods [including Annex 2C]...or Seventeen (Intellectual Property Rights) [which includes article 17.10.4] is being nullified or impaired as a result of a measure that is not inconsistent with this Agreement.

This type of NVNB claim has thus rightly been considered an 'exceptional remedy,' closely restricted to upholding concessions made during negotiations, and one that should be approached with 'extreme caution' [51]. This is reflected in the fact that there have been less than ten disputes in the history of the GATT and WTO involving NVNB claims. The exceptional nature of the NVNB claim is also highlighted by Article 26 of the DSU which provides that the complaining party carry the burden of providing a "detailed" justification in support of its complaint [52].

NVNB claims aim to ensure the obligation to act in good faith, with a particular duty of 'transparency and openness,' applies to the negotiating process, as Articles 31 and 32 of the *Vienna Convention on the Law of Treaties *(VCLT) (incorporated in the AUSFTA via article 21.9.2) do with respect to the implementation of the text of a treaty [53]. In NVNB disputes, the inquiry to be made by a dispute resolution panel is whether the complaining party was induced into error by the other treaty Party about a fact or situation that the former could not reasonably have foreseen [54].

The GATT and WTO dispute settlement panels have identified certain key elements that an NVNB complainant must establish in order to gain relief: (1) the application of a measure by a party; (2) a benefit was reasonably expected to accrue under the agreement or as a result of a fact or situation represented by a party during negotiations; (3) nullification or impairment of that benefit as a result of the application of the measure; (4) the nullification or impairment could not reasonably have been expected at the time of the agreement.

A necessary fifth element, however, is that the NVNB claim must specify breach of a clearly defined treaty obligation, rather than breach of a constructive ambiguity. Constructive ambiguities are the textual manifestations of negotiating truces. They are inherently ill-defined and of uncertain scope and application: an obvious example being valuing of pharmaceutical 'innovation' in Annex 2C.1 of the AUSFTA, where Australian and the US negotiators inserted competing definitions. Any panel failing to give adequate respect to the principle that NVNB claims presumptively only apply to manifestly explicit obligations, would be countenancing indefinitely protracted negotiations with destabilizing unpredictability and break down of good faith in interpretation of international trade obligations.

## Conclusion

The Canadian NOC linkage regulations are an ongoing source of controversy not only domestically in Canada between the generic and brand name sectors of the pharmaceutical industry, but also between the US pharmaceutical industry and the Canadian government. In financial terms, if the generic industry is to be believed, these brand name pharmaceutical industry-sponsored, 'pro-evergreening' regulations have probably added hundreds of millions of dollars to the Canadian drug bill since 1993 when they were first put into place and may be partly responsible for the increase in co-payments and deductibles in publicly funded provincial drug plans. Furthermore, if the dispute around their enforcement between Canada and the US continues to escalate there is the potential lead for the US to impose trade sanctions against Canada. Canada has to some extent ameliorated the impact of these regulations through the supervision of a dedicated patented list by the Office of Patented Medicines and Liaison.

Despite multinational pharmaceutical industry protestations to the contrary, article 17.10.4 of the AUSFTA is probably intended, and is likely, to pose similar 'evergreening' problems to Australia. Pharmaceutical policy reforms since the AUSFTA (as the result of strong lobbying by Medicines Australia) have been almost uniformly to the detriment of generics manufacturers and to the benefit of brand name companies, despite the total dominance of foreign interests in the latter camp. Vigorous enforcement of the anti-evergreening legislation in Australia, though entirely justifiable under both TRIPS and the AUSFTA, may lead to US threats of trade retaliation. The issue for Australian officials may be whether they have the nerve to call the US bluff (over how to value innovation and what were the parties respective legitimate expectations) and initiate dispute resolution procedures in a WTO forum, before less favorable AUSFTA mechanisms are invoked.

One of the most important lessons for Australia from the Canadian experience with 'linkage' evergreening may be the need to create a multidisciplinary regulatory oversight body such as the Office of Patented Medicines and Liaison. The Koreans seem to be heading in this direction. Another may be the importance of not being backward in preparing for and initiating WTO dispute resolution proceedings over related pharmaceutical policy issues. If the New Zealand government manages to 'carve out' 'linkage evergreening' from its TGA-inherited Trans-Tasman Therapeutic Products Agency obligations it may paradoxically have created a reason for generic firms to relocate in that nation. The recent mooted agreement over 'fast track' trade agreement approvals between the US Trade Representative and US Democrats may see 'linkage evergreening' scrapped from future US bilateral trade agreement negotiations. Whether this will lead to a reconsideration of its place in the KORUSFTA and AUSFTA remains to be seen, but is devoutly to be wished by all interested in sustainable industry profits linked to improved public health outcomes.
